# The Correlation Between MicroRNAs and Diabetic Retinopathy

**DOI:** 10.3389/fimmu.2022.941982

**Published:** 2022-07-25

**Authors:** Xin Zhao, Feng Ling, Guang wei Zhang, Na Yu, Jing Yang, Xiang yang Xin

**Affiliations:** ^1^Department of Ophthalmology, Inner Mongolia Baogang Hospita, Baotou, Inner Mongolia, China; ^2^Department of Cardiology, The First Affiliated Hospital of Baotou Medical College, Inner Mongolia University of Science and Technology, Baotou, Inner Mongolia, China; ^3^Department of Scientific research, Inner Mongolia Baogang Hospita, Baotou, Inner Mongolia, China; ^4^Department of Biology, Inner Mongolia University of Science and Technology Baotou Medical College, Baotou, Inner Mongolia, China

**Keywords:** micro ribonucleic acids (miRNAs), diabetic retinopathy (DR), inflammation, oxidative stress, neurodegeneration

## Abstract

Micro ribonucleic acids (miRNAs), as a category of post-transcriptional gene inhibitors, have a wide range of biological functions, are involved in many pathological processes, and are attractive therapeutic targets. Considerable evidence in ophthalmology indicates that miRNAs play an important role in diabetic retinopathy (DR), especially in inflammation, oxidative stress, and neurodegeneration. Targeting specific miRNAs for the treatment of DR has attracted much attention. This is a review focusing on the pathophysiological roles of miRNAs in DR, diabetic macular edema, and proliferative DR complex multifactorial retinal diseases, with particular emphasis on how miRNAs regulate complex molecular pathways and underlying pathomechanisms. Moreover, the future development potential and application limitations of therapy that targets specific miRNAs for DR are discussed.

## 1 Introduction

Micro ribonucleic acids (miRNAs), a family of post-transcriptional gene inhibitors commonly existing in animals, are widely involved in regulating gene expression in various environments (1). The advent of multicellular life was accompanied by new gene categories, including the most prevalent one: genes that encode miRNAs. Multiple articles on miRNAs have been published (about 136,000). These studies have determined that the genes encoding miRNAs can be involved in cell growth, apoptosis, fibrosis, hypertrophy, and senescence (2). Following the first demonstration of the role of miRNAs in chronic lymphocytic leukemia by Calin et al. in 2002 (3), several disease-related dysregulated miRNAs have been reported, such as those in cancer (4), diabetes (5), and cardiovascular diseases (6). To date, deciphering the exact nature of the molecular mechanisms associated with miRNAs in healthy and disease conditions remains challenging in related research. Recently, the efforts of some researchers on the role of miRNAs in diabetic retinopathy (DR) have achieved certain results (7). The main focus of this review is on the roles of miRNAs in DR, diabetic macular edema (DME), and proliferative DR (PDR) as well as their cellular and molecular mechanisms.

## 2 Biological Processes and Functions Of miRNAs

MicroRNAs are a category of short non-coding RNAs with a length of about 19 to 25 nucleotides. MicroRNA genes are transcribed by RNA polymerase II into single-stranded primary miRNA (pri-miRNA) with a length of more than 1 kb in the cell nucleus; then, they are processed and matured by a microprocessor complex composed of nuclear RNase III Drosha and cofactor DGCR8 (8). RNase III Drosha cleaves pri-miRNA and releases hairpin-like precursor miRNA (pre-miRNA) containing 65 bases. Pre-miRNA is transported to the cytoplasm, where the Dicer endonuclease cleaves it into 21 nucleotides of small double-stranded RNA near both ends of the ring (9). One strand of the obtained double-stranded RNA forms into a complex with a protein of GW182 (from the Argonaute [AGO] family) (10). This complex is called the miRNA-induced silencing complex (miRISC). In most cases, the nucleotides located at positions 2–7 in miRNA are in the “seed” region, which binds to the 3’ untranslated region nucleotides in messenger RNA (mRNA). The miRISC proteins take effect by recruiting factors that inhibit mRNA translation and promote mRNA deadenylation (11). Some miRNAs completely bind in the gene coding region, inducing the AGO protein to cleave mRNA (12).

Thanks to the development of miRNA sequencing technology, it has been found that each species in the animal kingdom has hundreds of annotated miRNA genes, and the number of miRNA genes recorded by humans is currently 556 (13). MicroRNAs can be divided into different families according to their mature sequences. MicroRNAs in a family can share the same sequence, but most miRNAs only share their seed sequences (14). About 300 miRNAs in humans belong to 177 different miRNA families (14). After finding evidence of the widespread existence of miRNAs in the entire animal kingdom, it was discovered that they guide many important processes related to cell growth, apoptosis, differentiation, metabolism, and immune response (15). During the occurrence and development of DR, miRNAs are involved in DR-related microvascularization, reverse dyslipidemia, and slowing DR progression (16). Moreover, they may be used in DR treatment strategies.

## 3 Expression of miRNAs in DR

The traditional belief that miRNAs are cell-autonomous was overturned in 2007 by discovering that miRNAs can be secreted into the circulatory system (17). The application of circulating miRNA mapping in diagnosing human disease has become a field of interest and a candidate biomarker in obesity, type 2 diabetes, atherosclerosis, and other diseases (18). **Table 1** summarizes the literature review on the expression of miRNAs in DR.

**Table 1:** Circulating MiRNA Biomarkers of DR.

miRNA	Tissue/cell	Expression	Reference
miR-190a-5p	Serum of DR patients	Up-regulation	(19)
miR-17-3p	Serum of DR patients	Down-regulation	(20)
miR-126	Serum of DR patients	Up-regulation	(21)
miR-210	Serum of DR patients	Up-regulation	(22)
miR-27b	Serum of DR patients	Up-regulation	(23)
miR-21	Plasma of PDR patients	Up-regulation	(24)
miR-29b	Plasma of DR patients	Down-regulation	(25)
miR-93	Plasma of DR patients	Up-regulation	(26)
miR-221	Peripheral blood endothelial progenitor cells cultured *in vitro*	Up-regulation	(39)
miR-216a	Human retinal microvascular endothelial cells	Down-regulation	(27)
miR-203a-3p	Rats retinal vascular endothelial cells	Down-regulation	(40)
miR-200b-3p	Rats retinal vascular endothelial cells	Down-regulation	(41)
miR-106a-5p	Circulating extracellular vesicles	Up-regulation	(43)
miR-150-5p	Circulating extracellular vesicles	Down-regulation	(28)
miR-15a	Circulating extracellular vesicles	Up-regulation	(29)
miR-20a-5p	Circulating extracellular vesicles	Down-regulation	(30)
miR-222	Circulating extracellular vesicles	Down-regulation	(31)
miR-199a-5p	Vitreous humor of PDR patients	Down-regulation	(32)
miR-204	Vitreous humor of PDR patients	Down-regulation	(33)
let-7a	Vitreous humor of PDR patients	Up-regulation	(34)
miR-16	Vitreous humor of PDR patients	Down-regulation	(35)
miR-92	Vitreous humor of PDR patients	Down-regulation	(36)

### 3.1 Serum

In the small-sample cohort study by Li et al., five miRNAs were found to be differentially expressed in the serum samples of the DR and non-DR (NDR) groups: the expression of miR-190a-5p was upregulated, and the expressions of miR-4448, miR-338-3p, miR-485-5p, and miR-9-5p were downregulated (19). According to another study on the expression of miR-20b and miR-17-3p in serum of the miR-17 family, the level in patients with DR was significantly lower than in healthy people (20). In a recent study involving 47 patients with type 2 diabetes, Tamir et al. found that 16 candidate miRNAs (miR-423, miR-486-3p, miR-320a-3p, miR-320p, miR-200b-3p, miR-221-3p, miR-146a-5p, miR-183-5p, miR-122-5p, miR-126-5p, miR-30p, miR-93-5p, miR-21, miR-27b-3p, let7f-5p, and miR-16-2-3p) changed in the serum of patients with diabetes (37).Other studies showed that the expression of miR-126 (21), miR-210 (22) and miR-27b (23) in the serum of DR patients was up-regulated.

### 3.2 Plasma

Qing et al. validated the elevated expression levels of three miRNAs (miR-21, miR-181c, and miR-1179) in plasma and predicted their role in PDR (38). In a study by Jiang, it was found that the level of miR-21 increased in the plasma of patients with PDR (24). Some studies have shown that the level of miR-29b in the plasma of patients with DR was significantly lower than in patients with no DR (25), and the level of miR-93 was significantly higher in the DR group than in the NDR group (26).

### 3.3 Peripheral Blood Endothelial Progenitor Cells

Garcia et al. obtained early growth endothelial progenitor cells by culturing peripheral blood monocytes *in vitro*. They detected the expression of miR-221 in the peripheral blood mononuclear cells of 41 patients with type 1 diabetes mellitus (T1 DM) and DR and 35 patients with T1 DM without DR by reverse transcription-polymerase chain reaction and found that the expression of miR-221 in the DR group was significantly higher than in the NDR group (39).The study of Liu et al. showed that the expression of miR-216a in human retinal microvascular endothelial cells was down-regulated (27), while Han et al. found that the expression of miR-203a-3p (40) and miR-200b-3p (41) in retinal vascular endothelial cells of rats was also down-regulated.

### 3.4 Extracellular Vesicles

Extracellular vesicles (EVs) larger than 200 nm are called “microvesicles,” which germinate outward from the plasma membrane. Smaller EVs can germinate outward through the plasma membrane to secrete exosomes, which can also be formed by fusing multivesicular bodies (MVBs) with the plasma membrane (42). The inward germination of late endosomes forms MVBs through the endosomal membrane to form intraluminal vesicles. The fusion of the MVBs with the plasma membrane will release the internal vesicles into the extracellular environment in the form of exosomes. In a recent study, the DR pattern of the miRNA map in circulating EVs was different from the healthy control group. It was found that the expressions of miR-21-3p, miR-17-5p, miR-106a-5p, and miR-21 were upregulated (43), and the expressions of miR-150-5p, miR-342-3p, and miR-155-5p were downregulated (28). MicroRNAs in EVs seem to be involved in the development of DR, and miR-150-5p, miR-21-3p, and miR-30b-5p extracted from circulating EVs are considered biomarkers of DR prognosis (44). Some studies showed that in the circulating EVS, the expression of miR-15a was up-regulated (29), while the expression of miR-20a-5p (30) and miR-222 (31) was down-regulated.

### 3.5 Vitreous Humor

For ethical reasons, healthy people cannot be used as controls. The general practice is to use the vitreous humor obtained from non-diabetic patients undergoing vitrectomy of the idiopathic macular hole as controls, so there is not much information on expression profiling in the vitreous bodies of patients with DR. According to the research results of Friedrich et al., the expression levels of miR-20a-5p, miR-23b-3p, miR-142-3p, miR-185-5p, miR-223-3p, miR-362-5p, and miR-662 in the vitreous humor of patients with PDR were higher than the control group, while those of miR-199a-5p and miR-326 were significantly lower than the control group (32). Some studies have shown that the expression level of miR-21 in the vitreous humor of patients with PDR was increased, and the expression levels of miR-204 and let-7c were decreased (33). The expression levels of miR-15a, miR-320a, miR-320b, miR-93, miR-29a, and miR-423-5p were significantly increased in the vitreous humor of patients with PDR (45). A recent study showed that the expression level of let-7a in the vitreous humor of PDR patients was elevated (34), while the expression level of miR-16 (35) and miR-92 (36) decreased.

## 4 Significance of miRNAs in RETINAL DISEASES

The retina is an important photosensitive neural tissue and plays a vital role in the central nervous system. It stems from a part of the central nervous system in the diencephalon, comprising the internal sensory retina and retinal pigment epithelium (46). The process in which oxidative stress, inflammation and metabolic disorder affect the structure and function of the central nervous system could cause irreversible impairment such as retinal degeneration and dysfunction. MiRNAs can be used as a gene regulator to regulate multiple biological processes such as neuronal function, innate immune response, cell proliferation and pluripotency (47). Highly expressed in retinas, it is a biomarker or therapeutic target of retinal diseases.

### 4.1 Diabetic Retinopathy

DR is the most common complication of diabetes, and also the disease affecting vision the most across the world. The abnormal change of retinal vasculature causes the reduction of blood supply to the retina, damage to the retinal microenvironment and retinal metabolic disorder, thus affecting the structure and function of the retina and leading to the loss of vision (48). The pathogenesis of DR is still unclear, so it is imperative to develop new diagnostic and therapeutic techniques (49). The expression of miRNAs in DR is discussed in Section 3, and the mechanism of action of MiRNAs in the pathological pathway of RD will be introduced in detail.

### 4.2 Proliferative Vitreoretinopathy

Proliferative vitreoretinopathy (PVR) is a complex disease, and results in severe loss of vision through forming a contractile retinal membrane (50). It is one of the main complications of rhegmatogenous retinal detachment surgery. So far, there has been no drug verified to treat or prevent PVR. Hiroki et al. found that the expression of miR-148a was up-regulated in vitreous humor of patients with retinal detachment, which was significantly associated with the expression level of inflammatory cytokine FGF-2. Besides, they found that miR-199a-5p was involved in the pathogenesis of PVR through inhibiting Caveolin-1 protein in retinal pigment epithelia (51).

### 4.3 Age-Related Macular Degeneration

Age-related macular degeneration (AMD) is a complex degenerative and progressive disease related to aging and oxidative stress (52), and it is characterized by the degeneration of retinal pigment epithelia between the photoreceptor and choroidal capillaries. A recent study showed that epigenetic mechanisms such as miRNAs regulation of gene expression were associated with the pathophysiology of AMD (53). The study of ElShelmani et al. showed that miR-19a, miR-126 and miR-410 played a role through regulating VEGF signaling, apoptosis and neurodegenerative pathways (54). Shahriari et al. found that hsa-let-7a-5p in retinal pigment epithelia inhibited neural differentiation and promoted the differentiation of retina pigment epithelia by acting on MITF (55).

### 4.4 Glaucoma

Glaucoma is a neurodegenerative retinal disease that is characterized by progressive and gradual loss of retinal ganglion cells (RGCs) and the axons, also called glaucoma neurodegeneration. It can continue and progress, thus leading to complete loss of vision (56). In the chronic glaucoma mouse model, investigators found that the expression of miR-149 was up-regulated in RGCs, while the silence of miR-149 promoted RGCs activity by increasing the activation of PI3K/Akt signaling pathway (57). Peng et al. found that the down-regulation of miR-200a in retinal ganglion cells of mice with glaucoma could increase the apoptosis of ganglion cells and the inactivation of Müller cells through enhancing the activation of MAPK mitogen-activated protein kinase signaling pathway (58).

## 5 Roles of miRNAs in DR INFLAMMATION AND OXIDATIVE STRESS

**Figure 1:**
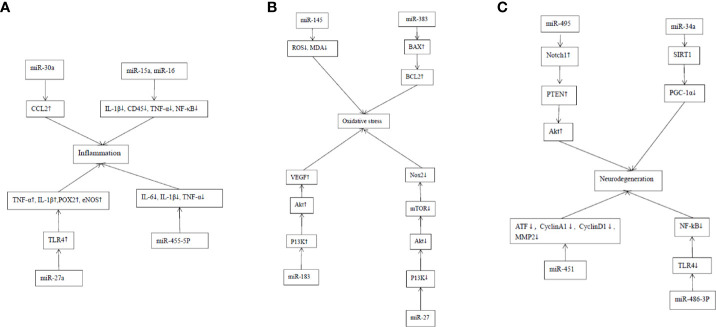
Mechanism of action of MiRNAs in DR inflammation, oxidative stress and neurodegeneration. **(A)** Mechanism of action of MiRNAs in DR inflammation. The targeting of miR-30a to CCL2 in retina enhances the phagocytosis of microglia in the retina. MiR-15a and miR-16 can inhibit the proinflammatory signaling pathway and stasis of retinal white blood cells in DR by inhibiting IL-1β, CD45, TNF-α and NF-κB. MiR-27a involves in the inflammatory response of DR by targeting TLR4 to stimulate the production of TNF-α and IL-1β as well as pro-inflammatory proteins including cyclooxygenase-2 and inducible nitric oxide synthase. The up-regulation of MiR-455-5p significantly attenuated the HG-stimulated inflammatory response by inhibiting the release of inflammatory cytokines, such as IL-6, IL-1β and TNF-α in ARPE-19 cells. **(B)** Mechanism of action of MiRNAs in the oxidative stress of DR. The overexpression of MiRNA-145 can reduce the levels of ROS and malondialdehyde (MDA) in cells, and increase the activity of superoxide dismutase, thus reducing hyperglycemia-induced oxidative stress and retinal endothelial cell apoptosis. MiRNA-383 can promote the production of ROS and induce apoptosis by increasing the expression of peroxidase redoxin 3 (PRDX3) and Bax/Bcl2. MiR-183 activates PI3K/Akt/VEGF signaling pathway, and increases CD34, endothelial nitric oxide synthase (eNOS) and ROS. MiR-27 inhibits Nox 2 signaling pathway by down-regulating P13K/AKT/mTOR, thus reducing the production of ROS. **(C)** Mechanism of action of MiRNAs in the neural degeneration of DR. MiR-495 affects the transmission of PTEN/Akt signal by targeting Notch1 and aggravates the damage to ganglion cells. MiR-34a promotes mitochondrial dysfunction and retinal microvascular endothelial cell senescence by inhibiting SIRT1/PGC-1α axis and mitochondrial antioxidants TrxR2 and SOD2. MiR-451a protects mitochondrial function through down-regulation and activation of transcription factor 2 (ATF2) and its downstream target genes CyclinA1, CyclinD1 and MMP2. The up-regulation of miR-486-3P can protect Müller cells in the HG state from oxidative stress, inflammation and apoptosis by inhibiting TLR4/NF-kB axis.

Diabetic retinopathy is characterized by chronic inflammation, oxidative stress, neurodegeneration, vascular leakage, neovascularization, and fibrosis. This section discusses the molecular interactions and signaling pathways during the regulation of miRNAs on the pathological processes of DR, including inflammation, oxidative stress, and neurodegeneration. The cellular and molecular mechanisms of miRNAs involved in these pathological processes come from cell culture and experimental animal models and are valuable for finding potential therapeutic targets for DR. **Table 2** summarizes the research progress of miRNAs and their target molecules in the pathogenesis of DR (**Figure 1**).

**Table 2:** Research progress of MiRNAs and their target molecules in the pathogenesis of DR.

miRNA	Possible signaling pathways	Pathogenic function	Reference
miR-15a and miR-16	Lacking it can increase retinal leukostasis and IL-1β, CD45, TNF-α and NF-κB	It can inhibit the pro-inflammatory signaling pathway and retinal leukostasis Participate in the inflammatory response of DR	(59)
miR-27a	Inhibiting it can stimulate the production of TNF-α and IL-1β as well as pro-inflammatory proteins including cyclooxygenase-2 and inducible nitric oxide synthase by targeting TLR4	Participate in the inflammatory response of DR	(60)
miR-455-5p	By inhibiting the release of inflammatory cytokines, such as IL-6, IL-1β and TNF-α, in ARPE-19 cells	Significantly attenuate HG-stimulated inflammatory response	(61)
miRNA-145	Reduce the levels of ROS and MDA in cells and increase the activity of superoxide dismutase	Reduce hyperglycemia-induced oxidative stress and retinal endothelial cell apoptosis	(62)
miRNA-383	Promote the production of ROS and induce apoptosis by increasing the expression of PRDX3 and Bax/Bcl2	Promote the development of DR	(24)
miR-183	Inhibit BTG1, activate PI3K/Akt/VEGF signaling pathway, and increase CD34, eNOS and ROS	Promote the development of DR	(82)
miR-27	Inhibit Nox 2 signaling pathway by down regulating P13K/AKT/mTOR, thus reducing the production of ROS	Slow the development of DR	(63)
miR-34a	Promote mitochondrial dysfunction and retinal microvascular endothelial cell senescence by inhibiting SIRT1/PGC-1α axis and mitochondrial antioxidants TrxR2 and SOD2	Promote the development of DR	(64)
miR-451a	Protect mitochondrial function through down regulation to activate ATF2 and its downstream target genes CyclinA1, CyclinD1 and MMP2	Slow the development of DR	(65)
miR-486-3P	Up-regulation of it can protect Müller cells in the HG state from oxidative stress, inflammation and apoptosis by inhibiting TLR4/NF-kB axis	Slow the development of DR	(66)
miR-320a	It can promote the internalization of aquaporin-4 (AQP4), thus reducing the edema of Müller cells under hypoxic stress	Slow the development of DR	(67)
miR-30a	Activate retinal microglia in an NLRP3-dependent manner	Promote the development of DR	(68)

### 5.1 Inflammation

MiRNAs can interact with a variety of inflammatory mediators, which plays an important role in the diversity of signal reactions in the process of DR development. MCP-1, also known as chemokine ligand 2 (CCL2), from the CC family, is a classical chemokine from the CC family. It leads to the progression of many diseases through the main mechanism of the migration and infiltration of inflammatory cells (such as monocytes, macrophages and other cytokines) at the site of inflammation. Murinello et al. showed that the targeting of miR-30a to CCL2 in retina enhanced the phagocytosis of microglia in the retina (69).

The key inflammatory response process is the adhesion and migration of peripheral blood leukocytes to vascular endothelial cells. Leukocyte retention may lead to retinal vascular leakage, enhancing retinal pathogenic effects. Studies have found that the cell adhesion molecule (CAM) increases in extraocular and retinal vessels in DR, resulting in increased leukocyte adhesion, vascular leakage, capillary nonperfusion, and endothelial cell injury (70). The increase of cytokines, chemokines, adhesion molecules, VCAM-1, and VEGF activates NF-κB, causing the leukocytes to adhere directly to vascular endothelial cells, increasing vascular permeability and damaging endothelial cells by releasing free radicals, enzymes, and cytokines (71). Recent studies have shown that the level of the cell signaling regulator pSTAT3 is elevated in circulating bone marrow monocytes from patients with diabetes and streptozotocin mice, resulting in a specific loss of SOCS3, which leads to leukostasis and capillary exfoliation increase (61). According to Ye et al., miR-15a and miR-16 could inhibit the pro-inflammatory signaling pathway and retinal leukostasis in DR, and the lack of these two miRNAs would increase retinal leukostasis, IL-1β, CD45, TNF-α, and NF- κB (59).

MicroRNAs are not only involved in the recruitment of immune cells but also regulate macrophage inflammatory immune signaling pathways at the site of injury. As an important pathogenic factor of DR, retinal hypoxia can activate macrophages, immune cells, and retinal microglia to release TNF-α, IL-8, VEGF, and MCP-1, leading to retinal ischemia and stimulating the production of VEGF and erythropoietin. Experiments have proven that inhibiting miR-27a can stimulate the production of TNF-α and IL-1β as well as pro-inflammatory proteins, including cyclooxygenase-2 and inducible nitric oxide synthase, by targeting toll-like receptor 4 (TLR4), thus participating in the inflammatory response of DR (60). In addition, the upregulation of miR-455-5p significantly attenuated the high glucose (HG)-stimulated inflammatory response by inhibiting the release of inflammatory cytokines, such as IL-6, IL-1β, and TNF-α in arising retinal pigment epithelial (19) cells (61). In conclusion, miRNAs are closely related to the inflammatory response, which plays a vital role in the pathological process of retinal vasculopathy, especially by mediating the interaction between immune and endothelial cells and the extracellular matrix by inducing the inflammatory signaling pathway.

### 5.2 Oxidative Stress

Metabolic abnormalities caused by hyperglycemia can lead to excessive the production of reactive oxygen species (ROS). The accumulation of ROS leads to oxidative stress, which damages the inner and surrounding tissues of retinal vessels. Finally, it leads to DR. There are four classical mechanisms of vascular injury caused by hyperglycemia (72). The association of miRNAs with oxidative stress in DR has been confirmed (73). In the following section, the action mechanism and research progress of miRNAs in DR oxidative stress are discussed in detail from four aspects.

#### 5.2.1 Increase of Polyol Pathway Flux

Hyperglycemia induces the activation of the polyol pathway and increases glucose content in cells. Aldose reductase (AR) reduces glucose to sorbitol, and sorbitol dehydrogenase oxidizes it to fructose. In this process, nicotinamide adenine dinucleotide phosphate (NADPH) and glutathione (GSH) are consumed to produce ROS, and the subsequent NADH oxidation is increased during the conversion of sorbitol to fructose. Aldose reductase, as the key rate-limiting enzyme in the polyol pathway, promotes the expressions of inflammatory factors TNF-α and NF-κB. The inhibition of glyceraldehyde-3-phosphate dehydrogenase contributes to the production of dihydroxyacetone phosphate and the increase of protein kinase C (PKC) and advanced glycation end products (AGEs), thereby inducing the increase of NADPH oxidase, the expression of inflammatory factors, and the decrease of endothelial nitric oxide synthase (eNOS) activation (74). The overexpression of miRNA-145 can reduce the levels of ROS and malondialdehyde, and increase the activity of superoxide dismutase, thus reducing hyperglycemia-induced oxidative stress and retinal endothelial cell apoptosis (62).

#### 5.2.2 Formation of AGEs and Increase of AGE Receptor Expression in Cells

The AGEs are advanced products of nonenzymatic glycation. Oxidized AGEs activate the receptor of AGEs (RAGE) to stimulate NADPH oxidase-1 and promote the production of ROS in diabetes (75). The AGEs can interact with scavenger receptors involved in AGE capture, removal, and degradation, as well as with pattern recognition receptors (i.e., RAGE) that initiate specific cell signaling (76). The AGE–RAGE interaction can activate a variety of signal transduction pathways, increase the expression of VCAM-1, macrophage inflammatory protein-1 matrix metalloproteinase 9 (MMP9), IL-1β, and TNF-α, and mediate leukocyte adhesion and the vascular inflammatory response, resulting in mitochondrial dysfunction and cell death (77). The RAGE ligands can activate the innate immune system response through TLR4 signaling and increased NF-κB transcription factor activity (78). Methylglyoxal, a precursor of AGE adducts, is elevated during hyperglycemia, which in turn promotes the expression of AGE, RAGE, and RAGE ligands (74). Studies have indicated that increasing the expression of miR-200c promotes the production of ROS (79). Additionally, MiRNA-383 can promote the production of ROS and induce apoptosis by increasing the expression of peroxidase redoxin 3 and Bax/Bcl2 (24).

#### 5.2.3 Pathway of Activating PKC

Protein kinase C is a serine/threonine-related protein kinase associated with diabetes. It has three subtypes: PKC-β, PKC-δ, and PKC-ζ. Protein kinase C-β can increase the expression of VEGF; aldose reductase activates PKC-δ; and the PKC-δ/p38 αMAPK pathway inhibits the apoptosis of pericytes and the formation of acellular capillaries induced by VEGF (80). Protein kinase C-ζ can be detected in endothelial cells and is involved in YYY- and VEGF-mediated cell proliferation and high permeability (81). Mir-183 upregulation inhibits B cell translocation gene 1, activates the PI3K/Akt/VEGF signaling pathway, and increases CD34, eNOS, and ROS (82). MiR-27 inhibits the Nox 2 signaling pathway by downregulating P13K/AKT/mTOR, thus reducing the production of ROS (63).

#### 5.2.4 Pathway of Activating Hexosamine

The increase of hyperglycemia-induced ROS transfers fructose-6-phosphate from the glycolytic pathway to the hexosamine pathway and produces UDP-N-acetylglucosamine by inhibiting the activity of glyceraldehyde-3-phosphate dehydrogenase. The rate-limiting enzyme is fructose-6-phosphate aminotransferase. In retinal neurons, the modification of glucosamine N-acetylglucosamine changes the neuroprotective effect of the insulin/Akt pathway (83). Studies have shown that hyperglycemia promotes N-acetylglucosamine acylation of NF-κB, which leads to retinal ganglion cell death (84). Hyperglycemia also reduces the expression of anti-inflammatory protein A20 through O-glucosamine-N-acetylation-dependent ubiquitination and proteasome degradation in response to inflammatory stimulation in VSMC and endothelial cells (85). Currently, there are no reports that miRNAs play a role in DR by activating the hexokinase pathway; this may be a future research area.

### 5.3 Neurodegeneration

Retinal tissue is composed of vascular tissues, neurons, and glial cells, all of which constitute the neurovascular unit. The occurrence of DR includes two interrelated components: diabetic retinal neurodegeneration (DRN) and diabetic retinal vasculopathy. The occurrence of DRN includes the apoptosis of ganglion and amacrine cells and the activation of Müller and microglia cells (MGs) (86). Previous studies have shown that miRNAs may be involved in the degeneration of Müller cells induced by DR (66). In the following section, the action mechanism of miRNAs in the occurrence and development of DR neurodegeneration and the application of miRNAs antagonists in neurodegeneration animal models will be described in detail.

#### 5.3.1 Apoptosis of Retinal Neurons (Retinal Ganglion and Amacrine Cells)

Many studies have shown that retinal neuronal dysfunction and apoptosis predate the development of vascular abnormalities in DR (87). Studies have also shown that the expressions of c-Fos/c-Jun, JNK, Bax, caspase-9, caspase-3, and Fluoro-Jade B apoptotic cell markers can be identified in the retina of patients with DR, and the activation of JNK/AP-1 signal transduction and the caspase-dependent cell death pathway in mitochondria can cause the degeneration of DR retinal ganglion cells (88). In the HG state, miR-495 may affect PTEN/Akt signal transmission by targeting Notch1 and aggravate the damage to ganglion cells (89). MiR-34a can promote mitochondrial dysfunction and retinal microvascular endothelial cell senescence by inhibiting the SIRT1/PGC-1α axis and mitochondrial antioxidants TrxR2 and SOD2 (64). It was found in experiments that overexpression of miR-451a may protect mitochondrial function by downregulating activating transcription factor 2 and its downstream target genes CyclinA1, CyclinD1, and MMP2, which provides a new perspective for the development of effective treatment for proliferative DR (65).

#### 5.3.2 Reactive Gliosis (Activation of MüLler Cells and Mgs)

As the primary macroglial cells in the retina, MGs are surrounded by neurons, extending from the ganglion cell layer to the photoreceptor inner ganglion region. In the early stage of DR, MG abnormalities, including increased expression of the glial fibrillary acidic protein, MG swelling, and activation of pro-inflammatory receptors in the microglia *via* P2X7 purines, can be observed (90). Microglia, including two activated phenotypes of pro-inflammatory (M1) state and anti-inflammatory (M2) state, is one of the components of neurovascular units. Pro-inflammatory microglia secrete IL-1β, IL-6, IL-8, and TNF-α cytokines, and anti-inflammatory microglia secrete IL-4, IL-10, IL-13, and transforming growth factor-β cytokines (91). It can be observed that microglia change from an anti-inflammatory state to a pro-inflammatory state before DR retinal neuron cell apoptosis. The increase of hyperglycemia-induced microglia AGEs stimulates the expression of TNF-α through ERK and NF-κB and induces VEGF expression in microglia through the ERK1/2-NF-κB signaling pathway (92). It has been shown in previous studies that upregulation of miR-486-3p protects MGs in the HG state from oxidative stress, inflammation, and apoptosis by inhibiting the TLR4/NF-kB axis (66). MiR-320a can promote the internalization of aquaporin-4 (AQP4), thus reducing the edema of MG under hypoxic stress (67). Under HG, miR-365 can promote retinal oxidative stress and microglial proliferation by regulating the expression of TIMP3, aggravating DR disease (93). MiR-30a activates retinal microglia in an NLRP3-dependent manner, thus promoting the progress of DR (68).

#### 5.3.3 Application of Mirnas Mimics and Antagonists in Neurodegeneration Animal Models

Streptozotocin-induced type 1 diabetic rats and type db/db2 diabetic mice are often used in research to simulate diabetic animal models. MiR-30a-3p synthesized in streptozotocin-induced type 1 diabetic rats can effectively alleviate retinal vascular dysfunction in rats (94). According to Chen et al., intravitreal injection of miR-21-5p inhibitors into the vitreous humor of db/db2 mice targets the peroxisome proliferator-activated receptor α and can reduce retinal inflammation (26).

## 6 Clinical Evidence of the Role of miRNAs IN DR

Diabetic retinopathy is a common microvascular complication of diabetes and the leading cause of blindness in adults. Diabetic retinopathy can be divided into non-PDR and PDR. Non-proliferative DR is clinically characterized by microaneurysm, intraretinal microvascular abnormality, and altered vascular permeability. Advanced PDR is characterized by hypoxia and angiogenesis. Due to the instability of these vessels, vitreous hemorrhage caused by vascular leakage may occur. Diabetic macular edema results from fluid accumulation in the inner and outer layers of the reticular retinal layer caused by changes in retinal blood flow. Diabetic retinopathy has been regarded as a vascular disease for many years, but it is now widely accepted that pathological processes such as oxidative stress, neurodegeneration, chronic inflammation, gliosis, and fibrosis play a vital role in the pathogenesis of early and advanced DR (95). The current treatments for DME include retinal laser photocoagulation, intravitreal VEGF inhibitor, intravitreal steroid, and vitrectomy. Unfortunately, each of these treatments has certain side effects. Retinal laser photocoagulation usually reduces the risk of moderate vision loss in patients with foveal DME but generally does not bring any visual gain (96). Frequent intravitreal injections of drug therapy (VEGF inhibitors and steroids) inconvenience most patients. Repeated use of VEGF inhibitors will increase the risk of fibrosis complications and tractive retinal detachment and may even cause neurodegeneration (97). Corticosteroids are associated with a high risk of cataract formation and elevated intraocular pressure (98). Therefore, efforts are being made to find new alternative drugs with long-term safety and effectiveness. Targeting specific miRNAs are viable treatment options for both DR and DME. The following section will focus on two promising methods to provide miRNA treatment.

### 6.1 Nuclease-Protected MiRNAs and Anti-MiRNAs

Nuclease has a poor affinity and easy degradation, but chemically-modified nucleic acids can overcome these shortcomings. Studies have shown that locked nucleic acid (LNA)-modified oligonucleotides have strong stability, nuclease resistance, high affinity, and specificity for complementary RNA or DNA oligonucleotides and have become a promising candidate for nucleic acid-based therapeutic strategy *in vitro* and *in vivo* (99).

Studies have shown that LNA anti-miRNAs can inhibit target molecules for up to 15 weeks in living animals, suitable for targeting DR candidate markers (100). The downregulation of miRNA activity can be achieved by adding nucleotide-based inhibitors bound to STA-B. A study by Huang et al. showed that the downregulation of miRNA activity could be achieved by adding nucleotide inhibitors that bind to STA-Bb to reduce the activity of miRNAs (101).

### 6.2 Delivery of MiRNAs to Target Tissues

Extracellular vesicles in organisms function to exchange nucleic acids, lipids, and proteins between cells. Such EVs represent a promising delivery vector for new drugs and gene therapy, with great potential for clinical applications, by manipulating EVs to load miRNAs *in vitro*, delivering them to target cells as drugs or for bioengineering purposes, or regulating the specificity of EVs of recipient cells as high-precision carriers. In practice, several miRNA delivery methods such as AAV vector, plasmid, piggyback expression vector, nanoparticles such as CC9 with tumor targeting and penetrating bifunctional peptides, and peptide nucleic acid-artificial peptide polymer combined with target nucleotide sequence are also used (102). Clinically, adding N-acetylgalactosamine to siRNA-based drugs can target miRNAs in specific tissues (103).

## 7 Conclusion and Prospects

In conclusion, miRNAs play an essential role in the key disease symptoms of DR, such as chronic inflammation, oxidative stress, and neurodegeneration. In addition, miRNAs are present in the circulating blood and vitreous humor, and they can regulate the above disease processes at multiple levels. They may play a role by interacting with various inflammatory factors (such as IL-1β, IL-6, TNF-α, IL-8, and MCP-1) and transcription factor NF-κB. Although the specific roles of miRNAs in the retina are not fully understood, they can be considered an essential part of the pathological signal transduction, and their role may have a therapeutic effect on DR. Nuclease-protected miRNAs and anti-miRNAs can be used to target DR candidate markers with the potential to exert broader biological effects. In clinical studies, gene therapy delivery vehicles, such as EVs, AAV vectors, plasmids, piggyback expression vectors, nanoparticles (including CC9), and artificial peptide polymers have shown favorable prospects. Despite the possibility of miRNAs in the treatment of multifactorial and complex ophthalmic diseases, including DR, given the fact that miRNAs have a broad impact on many biological functions and basic cell signal cascades, it is necessary to take into account the possible traps based on miRNA therapeutic targets, monitor the possible adverse reactions of miRNA therapy, and determine a safe therapy window.

## Author Contributions

Conception and design of the research: XZ and FL; Acquisition of data: GZ and NY; Analysis and interpretation of the data: FL; Statistical analysis: GZ and NY; Obtaining financing: XZ; Writing of the manuscript: XZ and FL; Critical revision of the manuscript for intellectual content: JY and XX. All authors contributed to the article and approved the submitted version.

## Funding

Health Science and Technology Plan of Inner Mongolia Autonomous Region Health Commission (No.202201522).

## Conflict of Interest

The authors declare that the research was conducted in the absence of any commercial or financial relationships that could be construed as a potential conflict of interest.

## Publisher’s Note

All claims expressed in this article are solely those of the authors and do not necessarily represent those of their affiliated organizations, or those of the publisher, the editors and the reviewers. Any product that may be evaluated in this article, or claim that may be made by its manufacturer, is not guaranteed or endorsed by the publisher.

## Abbreviation

MiRNAs, MicroRNAs; DR, Diabetic Retinopathy; DME, Diabetic Macular Edema; PDR, Proliferative Diabetic Retinopathy; miRNA, pre-miRNA; AGO, Argonaute; miRISC, miRNA-Induced Silencing Complex; UTR, three prime untranslated region; RT-PCR, Reverse Transcription-Polymerase Chain Reaction; MVB, Multivesicular Body; EV, Circulating extracellular vesicles; TF, Transcription Factor; PVR, Proliferative Vitreoretinopathy; AMD, Age-related Macular Degeneration; RGCs, Retinal Ganglion Cells; IL-1β, Interleukin-1β; IL-6, Interleukin-6; TNF-a, Tumor Necrosis Factor- a; IL-8, Interleukin-8; MCP-1, Monocyte Chemoattractant Protein-1; NF-κB, Nuclear Factor-κB; CAM, Cell Adhesion Molecule; EPO, erythropoietin; TLR4, Toll like receptor 4; ROS, reactive oxygen species; AR, Aldose Reductase; GAPDH, glyceraldehyde-3-phosphate dehydrogenase; DHAP, dihydroxyacetone phosphate; eNOS, Nitric oxide synthase; MDA, malondialdehyde; RAGE, Receptor for Advanced Glycation End products; MIP-1, Macrophage Inflammatory Protein-1; MMP 9, Matrix metalloproteinase 9; PRDX 3, Peroxidase redoxin 3; ox-LDL, Oxidized low density lipoprotein; BTG1, B cell translocation gene 1; GFAT, Glutamine:fructose-6-phosphate aminotransferase; GlcNAc, Glucosamine N-acetylglucosamine; DRN, Diabetic Retina Neuropathy; DRV, Diabetic RetinaVessel; ATF2, activating transcription factor 2; MG, Müller glia cells; GCL, Ganglion Cell Layer; GFAP, Glial Fibrillary Acidic Protein; AQP4, Aquaporin-4; PPAR-a, Peroxisome Proliferator-Activated Receptor-α; NPDR, Non-proliferative Diabetic Retinopathy; IRMA, Intraretinal Microvascular Abnormalities; LNA, Locked Nucleic Acid; Gal-NAC, N-Acetyl-galactosamine.
